# Energy budgets of captive Chinese pangolins (*Manis pentadactyla*)

**DOI:** 10.1093/conphys/coad049

**Published:** 2023-07-13

**Authors:** Hongmei Yan, Fuyu An, Quansheng Liu, Qingsheng Chi, Kai Wang, Xuelin Xu, Yingjie Kuang, Zhidong Zhang, Yan Hua

**Affiliations:** Guangdong Provincial Key Laboratory of Silviculture, Protection, and Utilization, Guangdong Academy of Forestry, 233 Guang Shan Yi Road, Tian He District, Guangzhou 510520, China; Guangdong Provincial Key Laboratory of Silviculture, Protection, and Utilization, Guangdong Academy of Forestry, 233 Guang Shan Yi Road, Tian He District, Guangzhou 510520, China; Guangdong Key Laboratory of Animal Conservation and Resource Utilization, Guangdong Public Laboratory of Wild Animal Conservation and Utilization, Institute of Zoology, Guangdong Academy of Sciences, 105 Xingang West Road, Haizhu District, Guangzhou 510520, China; College of Biology and Agriculture, Zunyi Normal University, Ping'an Avenue, Xinpu New District, Zunyi 563006, China; Guangdong Provincial Key Laboratory of Silviculture, Protection, and Utilization, Guangdong Academy of Forestry, 233 Guang Shan Yi Road, Tian He District, Guangzhou 510520, China; Guangdong Provincial Key Laboratory of Silviculture, Protection, and Utilization, Guangdong Academy of Forestry, 233 Guang Shan Yi Road, Tian He District, Guangzhou 510520, China; Guangdong Provincial Key Laboratory of Silviculture, Protection, and Utilization, Guangdong Academy of Forestry, 233 Guang Shan Yi Road, Tian He District, Guangzhou 510520, China; Guangdong Provincial Key Laboratory of Silviculture, Protection, and Utilization, Guangdong Academy of Forestry, 233 Guang Shan Yi Road, Tian He District, Guangzhou 510520, China; Guangdong Provincial Key Laboratory of Silviculture, Protection, and Utilization, Guangdong Academy of Forestry, 233 Guang Shan Yi Road, Tian He District, Guangzhou 510520, China

**Keywords:** resting metabolic rate, respirometry, Manis pentadactyla, daily energy expenditure, Chinese pangolin

## Abstract

The Chinese pangolin is an endangered species, and *ex situ* conservation and captive rescue are important conservation measures. This requires reliable information on nutritional energy requirements and expenditure characteristics. However, we lack sufficient knowledge of their energy physiology to determine their energy requirements for maintenance and growth. An open-flow respirometry system was used to measure the resting metabolic rate (RMR) and the daily energy expenditure (DEE) of Chinese pangolins (*Manis pentadactyla*), and the dietary digestive energy was measured. The average RMR in Chinese pangolins was 3.23 ml O_2_ kg^−1^ min^−1^ at an ambient temperature (*T*_a_) of 24.5–30°C, which was only 73.0% of the expected value based on body mass (BM). The average DEE values were 744.9 kJ day^−1^ in animals with BM >3 kg and 597.3 kJ day^−1^ in those with BM <3 kg, which were only 52.4% and 60.6% of the predicted values, respectively. The RMR and DEE levels of the Chinese pangolin were lower than those of similar-sized eutherian mammals and close to those of anteaters. These characteristics suggest that the Chinese pangolin has a low demand for energy in its diet. Although metabolic level data alone cannot be used to calculate the energy requirements of each Chinese pangolin, we believe they can provide a tangible reference for the relocation of Chinese pangolins. These results provide a scientific basis for future research on the physiology and ecology of endangered wildlife such as the Chinese pangolin.

## Teaser

We measured the metabolic level of Chinese pangolins using breath calorimetry, demonstrating that Chinese pangolins have a low metabolic rate. Compared to other animals, Chinese pangolins require less energy to survive in captivity. This study lays the foundation for better *ex situ* conservation of Chinese pangolins.

## Introduction

The Chinese pangolin is a typical anteater, preying primarily on termites and ants in the wild (Sweeney, 1956; Yang *et al.,* 1999; Min *et al.,* 2020; Mahmood *et al.,* 2021), and is a member of the order Pholidota and family Manidae. It is listed in CITES appendix I and is ranked in the first-class level of protection for species in China (Hua *et al.,* 2020). The Chinese pangolin (*Manis pentadactyla*) is one of the most threatened and trafficked mammals in the world (Mohapatra *et al.,* 2015). Human interference and habitat fragmentation are important reasons for the decline in wild populations (Gao *et al.,* 2022). With the exhaustion of wild populations, *ex situ* conservation has become one of the most important methods to protect Chinese pangolins from extinction (Hua *et al.,* 2015). Many initiatives are being taken to boost their populations, such as captive breeding, returning captive-bred animals to their natural habitats and conservation studies of wild habitats. However, there still exist many technical barriers in *ex situ* environments because of the pangolin’s specialized physiology and dependence on natural systems (Lim, 2008; An *et al.,* 2023). The lack of data on the characteristics of the energy budget of Chinese pangolins is one of the factors affecting the healthy maintenance and growth of captive populations.

Animals can be viewed as open energy converter systems (Bozinovic, 1992). Every biological structure has an energy content; every physiological function and activity requires energy metabolism and transformation (Carey, 2012). An animal should meet the dynamic balance of its energy budget in most stages of life. The metabolic rate of the pangolin has been assessed in several studies, but only resting metabolic rates (RMRs) have been reported for the Chinese pangolin (Heath and Hammel, 1986), and their daily energy expenditure (DEE) has not been measured, and their metabolic levels have not been analysed. As a typical anteater, the metabolic level of Chinese pangolins should be close to that of other myrmecophagous mammals, such as anteaters and armadillos, and lower than that of other eutherian mammals. The burrowing nature of the Chinese pangolin (Bao *et al.,* 2013) makes its metabolic level lower than that of terrestrial arboreal animals (McNab, 1979; Lovegrove, 1986; Bennett and Spinks, 1995). The Chinese pangolin is mainly distributed in Southeast Asia and other areas and used to be widely distributed south of the Yangtze River in China (Wang *et al.,* 2022); it should have a higher metabolic level than other anteaters and pangolins distributed in tropical areas. Therefore, we predict that the metabolic level of Chinese pangolins should be lower than that of eutherian mammals, close to that of anteaters, lower than that of arboreal and terrestrial pangolins and higher than that of tropical burrowing pangolins. In addition, there are relatively few studies on the metabolic levels and dietary digestive energy of the Chinese pangolin. Therefore, it is not possible to accurately estimate the energy budget of the Chinese pangolin under captive conditions.

The goal of this study is to reveal the energetic and metabolic characteristics and energy input and expenditure of Chinese pangolins through a comprehensive study and to provide a reference for the scientific formulation of feeding systems. In this study, we describe the open respirometry measurements of captive Chinese pangolins’ RMR and DEE. In addition, we tracked and documented daily activity, as well as changes in body weight, body temperature and digestion energy.

## Materials and methods

### Animals

Eight healthy Chinese pangolins used in this study were reared in the Guangdong Wildlife Monitoring and Rescue Center, China. They were rescued in Guangdong Province from 2019 to 2021.

The Chinese pangolins live alone in an enclosure, and their diet consists mainly of raw insect materials, such as ants, bread worms and earthworms, supplemented by natural foods such as termites. The enclosure is divided into two parts, inside and outside, with an inside area of approximately 5 m^2^, nesting boxes for activities and resting, climbing frames and other ample facilities. The Chinese pangolins were brought indoors when the weather outside was cold (temperatures lower than 24°C). The temperature (24–29°C) and humidity (50–60%) of the inside room were controlled and maintained by air conditioning (Media KFR-50GW, Guangdong, China). Outside, diverse environments such as grass, mounds, ponds and trees are used to create their habitat and support behavioural expression.

No animal was sacrificed during the experiment, and no damage was caused to the animal body. All pangolins were in good physical condition during the study period, exhibiting normal levels of activity, feeding and defecation. All animal experiments and data collection procedures were subject to approval by the Guangdong Academy of Forestry, and support and permission for conducting the experiments were received from the Wildlife Rescue Monitoring Center of Guangdong Province, China.

### Resting metabolic rate

Measurement of the RMR began in October–November 2022. Experience has shown that if Chinese pangolins are exposed to temperatures above 30°C or below 20°C for prolonged periods, they become heat/cold stressed and experience health problems. Moreover, according to the husbandry rules of the Wildlife Rescue Monitoring Center of Guangdong Province, Chinese pangolins should not be exposed to temperatures lower than 23°C or greater than 30°C. Therefore, in our experiment, we attempted to keep the maximum temperature at 30°C and the minimum temperature at 24°C.

Seven animals (three females and four males) were used (Table 1). One animal was examined at a time. Because Chinese pangolins are nocturnal, we conducted all experiments during daylight hours (0900–1800). Chinese pangolins were weighed before and after each experiment. The rectal temperature (taken in the anal opening to a depth of at least 50 mm) of the Chinese pangolins was measured after each experiment. Animals fasted for 12 h before the metabolic rate tests. Pangolin MP5 was pregnant during the experimental period and thus was not involved in the RMR experiment. One month before measurement, pangolin MP6 gave birth to a baby pangolin that died on the third day. Therefore, metabolic data were collected for this individual while she was in lactation.

**Table 1:** RMRs of Chinese pangolins measured in a metabolic chamber (M is male, and F is female)

Animal	Mass (kg)	Sex	Temperature (°C)	Body temperature (°C)	Rectal temperature (°C)	RMR O_2_ (ml kg^−1^ min^−1^)
MP1	4.737	M	28.5	29.2	32.5	3.20
MP2	6.172	M	30	32.3	33.5	3.22
MP3	3.974	F	29	32.5	33.1	3.23
MP4	4.643	M	27	31.1	33.2	3.58
MP6	3.452	F	25	29.7	33	4.82
MP7	4.076	M	24.5	31.5	32.5	3.12
MP8	4.381	F	27	30.4	32.7	3.04

We used a standard open-flow respirometry technique to determine the RMR of the Chinese pangolin. The respirometer consists of an acrylic respiration chamber (volume: 190.1 l; dimensions: 0.45 m × 0.65 m × 0.65 m) covered with a blackout cloth and placed in a room in which the temperature was controlled with air conditioning. One side of the chamber was a door made of acrylic with more than 10 evenly distributed 1-cm inlet holes and an exit hole 2 cm from the bottom of the breathing chamber on the side opposite to the inlet hole and connected to a silicone tube leading to a diaphragm pump (KLP04-320-12; Kamoer, Shanghai, China). The air sample was passed through a diaphragm pump to flow control metres (YJ-700CF-AIR-10SPLM, Nanningkongxin Industry, Guangxi, China) into the FOXBOX oxygen and carbon dioxide analyser (Sable Instruments International, Las Vegas, LV) at a rate of 3500 ml min^−1^. An iButton temperature logger (Maxim Integrated, San Jose, CA) was located on top of the respiratory chamber to measure and record the respiratory chamber temperature. Air was drawn from the chamber outlet hole by a pump at a rate of 3500 ml min^−1^ and passed through the desiccator into the FOXBOX respirometry system (Sable Instruments International) for analysis. The analyser uses nitrogen (99.999% N_2_) for zero calibration and a standard gas (20.6% O_2_, 0.6% CO_2_) (from Guangzhou Yinglai Gas Industry Company) to calibrate the oxygen and carbon dioxide values. After the analyser was started, it was preheated for 3 h to reach a stable state, and the room air baseline was measured after stabilization. The tubing was connected to the breathing chamber, the oxygen concentration passing through the breathing chamber was measured and the data were recorded every 5 s. Prior to the start of each measurement, animals were allowed to acclimatize in the breathing chamber for at least 1 h after becoming inactive (as evidenced by observation of animal behaviour via infrared camera). We calculated oxygen consumption (VO_2_) as the mass ratio rate (ml O_2_ kg^−1^ h^−1^) and selected 15 min of continuous stable data, discarding the first hour of data (Lighton, 2018). Oxygen consumption was calculated using equation (1):

(1) \begin{equation*} \mathrm{V}{\mathrm{O}}_2=\frac{\left(\mathrm{FR}\left(\left(\mathrm{Fi}{\mathrm{O}}_2-\mathrm{Fe}{\mathrm{O}}_2\right)+\mathrm{Fi}{\mathrm{O}}_2\left(\mathrm{Fi}\mathrm{C}{\mathrm{O}}_2-\mathrm{Fe}\mathrm{C}{\mathrm{O}}_2\right)\right)\right)}{\left(1-\mathrm{Fi}{\mathrm{O}}_2\right)} \end{equation*}

FR is the flow rate (STP), FiO_2_ is the input fractional concentration of O_2_ to the chamber, FeO_2_ is the excurrent fractional concentration of O_2_ from the chamber, FiCO_2_ is the input fractional concentration of CO_2_ to the chamber and FeCO_2_ is the excurrent fractional concentration of CO_2_ from the chamber (Chi and Wang, 2011).

### DEE and activity budget

Measurement of the DEE occurred in August–October 2021. A total of eight animals (four females and four males) were used in this test (Table 2).

**Table 2:** Chinese pangolins’ DEE and activity time were measured in a metabolic chamber (M is male, and F is female)

Animal	Mass (kg)	Sex	DEE (kJ day^−1^)	Activity Time (min day^−1^)
MP1	4.098	M	756.28	185
MP2	5.628	M	759.13	53
MP3	4.447	F	808.60	260
MP4	2.385	M	635.85	113
MP5	2.239	F	490.58	38
MP6	2.756	F	628.88	300
MP7	4.337	M	655.62	147
MP8	2.866	F	633.95	295

Pangolins with body weights less than 3 kg were tested in the chamber (volume: 190.1 l; dimensions: 0.45 m × 0.65 m × 0.65 m), and pangolins with body weights greater than 3 kg were tested in a different chamber (volume: 420 l; dimensions: 0.7 m × 0.5 m × 1.2 m). Before beginning each measurement, we allowed animals to acclimatize to the chamber for at least an hour, during which time the oxygen consumption reading stabilized and animals became inactive (activity was verified via an infrared camera). Each DEE measurement lasted for 26 h, and each animal was measured at least two to three times. The ambient temperature was maintained at 26.5 ± 1.0°C. Body weight was measured before and after each metabolic measurement. The animals were allowed to eat and drink ad libitum during the measurement period. Assuming that 1 ml of oxygen releases 20.1 J of energy, we converted VO_2_ to energy expenditure (Nagy, 1983).

We monitored and recorded the actions of the Chinese pangolins in a captive setting using infrared monitoring equipment. For 24 h, we watched and videotaped the pangolin continually, noting their actions at the end of each minute. These actions were divided into three categories: eating, sleeping and other activities. The Chinese pangolin’s active (feeding and engaging in other activities) and inactive (resting) times were then calculated.

### Digestible energy and body mass

The digestible energy measure was used for six animals because two animals (MP7 and MP8) had diarrhoea. This experiment began after the DEE measurement for each animal was finished, lasting 7 days. During the sampling weeks, animals were provided with pre-weighed food. Uneaten food and faeces were collected every morning and oven dried at 60°C to a constant mass. The gross energy (GE) content of the feed and faeces was determined with a 6200 Isoperibol Calorimeter (Parr Instrument Company, Moline, IL). The energy content of the dry diet was 21.75 kJ g^−1^. Energy intake and digestible energy were computed according to Song and Wang (2006). The intake and excretion of GE were calculated by multiplying the corresponding amount of DM in the GE content of the corresponding sample. The apparent digestibility of the GE was calculated as intake minus excretion using equation (2):

(2) \begin{equation*} \mathrm{DE}=\mathrm{DM}\times \mathrm{GE}-\mathrm{FM}\times \mathrm{GE} \end{equation*}

DM is the intake of dietary dry matter, GE is the matter of GE and FM is the faeces dry matter.

Body weight measurement was performed once each week over a period of 3 months using an electronic scale with an accuracy of 1 g.

### Statistical analysis

The data obtained in this study were analysed using SPSS 23.0. Linear regression analysis was used to identify the statistical significance of the association between RMR, body mass (BM) and other mammalian RMRs and DEEs. All data are presented as the mean ± SEM. *P* < 0.05 was considered statistically significant.

## Results

### Resting metabolic rate

**Figure 1:**
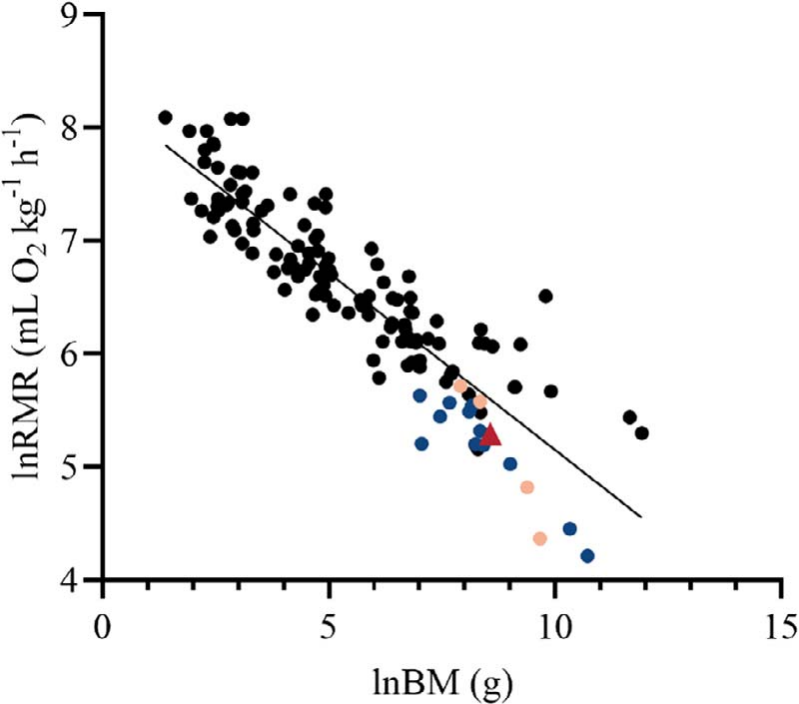
RMR by respirometry of terrestrial mammals [ln RMR (ml O_2_ kg^−1^ h^−1^)] plotted against BM [ln mass (g)]. Each point represents a different species (data from Hayssen and Lacy, 1985; Boyles *et al.,* 2020). The red triangle data point represents the Chinese pangolin (*M. pentadactyla*). The other pangolins were represented by the yellow points. The myrmecophagous animals like anteaters and armadillos were represented by the blue circle points.

The RMR of the Chinese pangolin ranged from 3.02 to 4.82 ml O_2_ kg^−1^ min^−1^ (Table. 1). The mean RMR of all animals except MP6 was 3.23 ml O_2_ kg^−1^ min^−1^ (193.8 ml O_2_ kg^−1^ h^−1^). The rectal temperature at the end of the metabolic measurement of the Chinese pangolins averaged 32.9°C. Compared with that of other terrestrial mammals, the RMR of the Chinese pangolins was only 73.0% of the expectation based on body size (Fig. 1).

### DEE and activity budget

**Figure 2:**
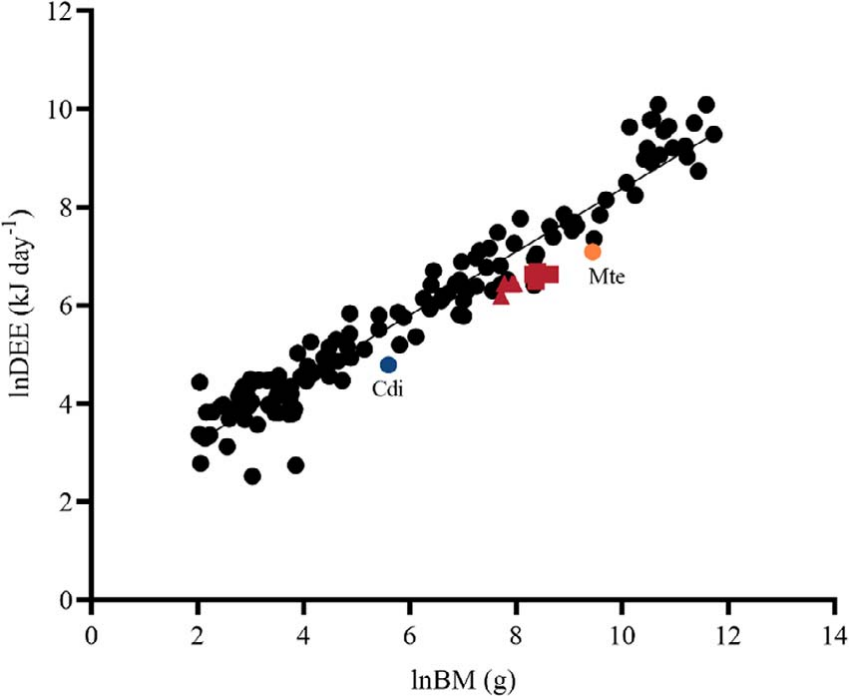
DEE of terrestrial mammals (by doubly labelled water) and DEE Chinese pangolins (by respirometry) [ln DEE (kJ day^−1^)] plotted against BM [ln mass (g)]. Each point represents a different species (data from Carbone *et al.,* 2007; Speakman and Król, 2010; Pontzer *et al.,* 2014; Nie *et al.,* 2015; Boyles *et al.,* 2020). The Chinese pangolin is represented by the red data point. Animals with BM >3 kg (*n* = 4) are represented by squares, and animals with BM < 3 kg (*n* = 4) by triangles. Mte, *M. temminckii* (ground pangolin); Cdi, *Cyclopes didactylus* (silky anteater).

Across individuals with an average BM of 4.63 kg, the DEE averaged 744.9 ± 27.8 kJ day^−1^ (±SEM) (*n* = 4 animals). Across individuals with an average BM of 2.56 kg, the DEE averaged 597.3 ± 30.8 kJ day^−1^ (±SEM) (*n* = 4 animals). Weight had a significant effect on DEE (*P* < 0.05) (Fig. 2). The DEE of 4.63-kg animals (obtained through respirometry) was only 52.4% of the value expected for terrestrial mammals based on body weight (DEE of doubly labelled water). The DEE of 2.56-kg individuals was 60.6% of the value expected based on BM (Fig. 2).

Under captive conditions, the average daily activity time of the Chinese pangolin was approximately 173.9 min (ranging from 38 to 300 min) (Table 2), with an average of 21.1 h of sleeping per day.

### Digestible energy and BM

**Figure 3:**
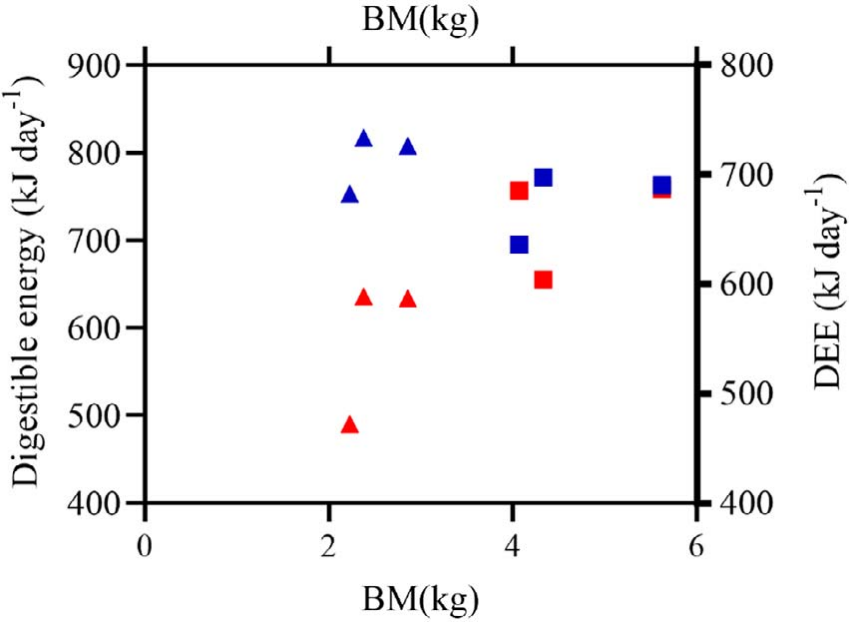
Energy budget of DEE animal. Animals with BM >3 kg (*n* = 3) are represented by squares, and animals with BM <3 kg (*n* = 3) by triangles. The red points represent DEE, and the blue points represent digestible energy.

Digestible energy for the animals with BM greater than 3 kg averaged 743.19 ± 19.7 kJ day^−1^ (±SEM) (*n* = 3), and that of the animals with BM less than 3 kg averaged 793.06 ± 16.2 kJ day^−1^ (±SEM) (*n* = 3) (Fig. 3).

**Figure 4:**
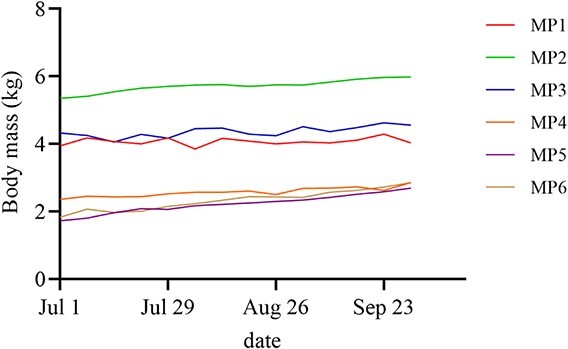
Body mass changes in six Chinese pangolins, which were measured DEE and digestible energy.

At the beginning and end of the experiment, the mean body weights of MP1, MP2 and MP3 were 4.53 ± 0.34 and 4.85 ± 0.47 kg (±SEM), respectively, and the mean body weights of MP4, MP5 and MP6 were 1.97 ± 0.16 and 2.8 ± 0.05 kg (±SEM), respectively (Fig. 4).

## Discussion

### RMR and DEE

Ambient temperature is an important factor that affects metabolism (Scholander *et al.,* 1953; Canterbury, 2002; Buckley *et al.,* 2012; Fei *et al.,* 2016). Mammals have a thermally neutral region, within which they exhibit the lowest RMR (Silva, 2006). Heath and Hammel (1986) suggested that temperatures of 25–30°C were in the thermally neutral zone (TNZ). In our experiment, there was no difference in metabolic rates at temperatures of 24 to 30°C. Therefore, those temperatures were within the TNZ, and the RMRs at those temperatures were similar to the basal metabolic rate (BMR).

Many factors, including diet, living habits, physical activity, anatomy and physiological characteristics, influence metabolism (Bozinovic, 1992; Farlow, 2005). Compared to the results obtained by Heath and Hammel (1986), who measured Chinese pangolin RMR (3.06 ml O_2_ kg^−1^ min^−1^), our testing results were slightly higher. This may be explained by different feeding conditions, different seasons or individual differences in animals at the time of measurement.

Our study compared the RMR and DEE of Chinese pangolins to those of other terrestrial mammals using a regression model. The RMR regression line equation is ln(RMR in ml O_2_·kg^−1^·h^−1^) = 8.281 − 0.3130[ln BM (g)] (*P* < 0.0001, *r*^2^ = 0.80). Compared with other terrestrial mammal DEEs, the regression line equation is ln(DEE in kJ day^−1^) = 1.950 + 0.6430[ln mass (g)] (*P* < 0.0001, *r*^2^ = 0.93). The metabolic level of the Chinese pangolin is lower than that of most mammals. At the same body weight, Chinese pangolin metabolic rates were close to those of the giant anteater (*Myrmecophaga tridactyla*), andean hairy armadillo (*Chaetophractus nationi*) and long-eared hedgehog (*Hemiechinus auritus*) and lower than those of carnivores and primates. The body temperature of the Chinese pangolin is also closer to that of an anteater, armadillo or hedgehog, which is approximately 32–35°C (Fowler, 1988; Kluyber *et al.,* 2021; Superina and Boily, 2007). Studies have shown that mammals that preferentially feed on termites and ants have very low BMRs (McNab, 1986a, 1986b). This may result from the ingestion of appreciable quantities of detritus with the food, which would dilute the energy present in the food (Stahl *et al.,* 2012) and may be correlated with the spatial and temporal distribution of the species and the chemical defence of termites and ants (McNab, 1980). In addition, the lower metabolic rate of myrmecophagous mammals may be accounted for by body temperature and its covariation with diet (Clarke *et al.,* 2010).

The Chinese pangolin RMRs were lower than those of the tree pangolin (*Manis tricuspis*) and Sudan pangolin (*Manis javanica*) and higher than those of the ground pangolin (*Manis temminckii*) and Indian pangolin (*Manis crassicaudata*). Fossorial and burrowing mammals generally have low metabolic rates compared with arboreal and semiarboreal mammals (McNab, 1979). According to Vleck (1979), digging a hole requires more energy than moving the same distance on a surface. The low BMRs of these animals may be related to an adaptation to conserve energy or the fact that they burrow to provide a stable temperature environment, decreasing heat consumption (Bao *et al.,* 2013). The tree pangolin and Sudan pangolin, which have higher metabolic levels, are mainly arboreal, while the Chinese pangolin, ground pangolin and Indian pangolin are all mainly burrowing. This supports our second hypothesis that the burrowing lifestyle of the Chinese pangolin could result in a lower metabolic rate. In contrast, when comparing the three burrowing pangolins, the higher metabolic level of the Chinese pangolin may be due to the colder climate of its range compared to that of the other two pangolins. Generally, mammals that live in cold climates have higher metabolic rates than those that live in warmer regions (McNab, 2008). The Chinese pangolin is mainly distributed south of the Yangtze River in China in a region with a temperate subtropical climate (Wu *et al.,* 2003, Zhang *et al.,* 2022), whereas the Indian pangolin and the ground pangolin are mainly found in regions with subtropical and tropical climates (Heath and Coulson, 1997, Phillips, 1980).

The body weights of Chinese pangolins with the same level of energy expenditure differed in this study. Chinese pangolins with less active time consumed less energy than more active Chinese pangolins of the same body size. Therefore, differences in activity time may contribute to the differences in energy expenditure of Chinese pangolins of the same body size. The open respirometry system measured lower energy expenditure than the double-labelled water method because of the greater restriction on the animal’s range of activity and activity intensity (Westerterp *et al.,* 1988; Westerterp, 2008). In addition, the activity time and intensity of the Chinese pangolin may have been reduced to different degrees due to space restrictions (Wang, 2000) and reduced foraging requirements under captive conditions. Therefore, the true metabolic rate of the Chinese pangolin in the wild needs to be further studied.

### Energy budgets

Animals require enough energy for maintenance, growth and reproduction (Hosey *et al.,* 2013). Energy intake and expenditure should be kept in dynamic balance. Positive energy balance is necessary for the completion of new tissue synthesis and embryonic development in animals during the anagenesis and gestation periods, respectively. However, positive energy balance primarily causes an increase in body fat content in mature nonbreeding animals. According to Chin *et al*. (2015), Chinese pangolins are classified as adults if they weigh more than 3.5 kg and subadults if they weigh less than 3 kg. In our study, the mean DEE of 744.9 kJ day^−1^ was similar to the daily digestible energy intake (743.2 kJ) of adult (BM >3.5 kg) Chinese pangolins, and the body weight remained stable. The mean DEE of subadult (<3 kg) Chinese pangolins was 597.3 kJ day^−1^, which was lower than the digestible energy intake (793.1 kJ day^−1^), and the body weight tended to increase significantly.

This suggests that the adult Chinese pangolins reached an energy balance and that the subadult Chinese pangolins maintained a positive energy balance at the feeding amount used in this study. To date, the determination of the feeding amount has typically been based on extrapolation from empirical data, and there is no scientific basis for its development. Experience has shown that low metabolic rates can lead to high energy availability in captivity, resulting in rapid weight gain or overweight animals. For example, the average weight of an individual Chinese pangolin in the wild is approximately 4 kg (Wu *et al.,* 2004), whereas the weight of Chinese pangolins in captivity recorded by Taiwanese Pangolin Raising Institutions reached 8 kg. In the growth period, for pangolins such as subadult Chinese pangolins, it is impossible to accurately determine whether they are obese or just growing based on their weight change. Therefore, we should refer to the animal’s energy consumption, activity time, energy from feed digestion and different growth periods to develop a scientifically based feeding system.

Conventionally, considerations for feeding wildlife have often focused on ingredient selection and diet composition, especially in the case of anteaters such as pangolins, but actual data are not often provided (Morford and Meyers, 2003), and foraging characteristics are not taken into account. The deviation of the metabolic rate of the Chinese pangolin from the average metabolic rate of anteaters suggests that we cannot simply use the conventional body weight index of 0.75 and 0.67 (Glazier, 2008) to estimate the energy expenditure of the Chinese pangolin. Differences in metabolic levels between animals with different activity levels suggest that feeding levels should be reduced appropriately for less active animals. Energy consumption should be met for animals in the growth phase to achieve a positive energy balance. However, the feeding amount should be adjusted according to the actual weight change in subadult Chinese pangolins. It is recommended to accumulate metabolic and weight gain data of subadults and improve the feeding regime to meet the optimal growth curve. Therefore, when developing a feeding regime, the actual feeding amount and feeding method should be adjusted based on regular body weight measurements after first meeting the energy balance requirements.

## Conclusion

Captive Chinese pangolins’ RMR and DEE are lower than those of eutherian terrestrial mammals. Their lower metabolic level may be mainly due to their specialized feeding habits, low body temperature and physiological adaptation to burrowing life. Their reduced dietary energy requirements should result in an appropriate adjustment of energy intake to their metabolic levels under captive conditions.

## Author Contributions

Methodology: Yan Hua, Hongmei Yan, Quansheng Liu and Qingsheng Chi.

Resources: Fuyu An, Xuelin Xu, Yingjie Kuang and Zhidong Zhang.

Data analysis and writing the article: Hongmei Yan, Quansheng Liu, Qingsheng Chi, Shichao Wei, Kai Wang and Yan Hua.

Supervision: Yan Hua.

## Conflict of Interest

All authors declare that there are no conflicts of interest.

## Funding

This study was funded by the National Key Program of Research and Development, Ministry of Science and Technology (No. 2022YFF1301500) and the Forestry Science and Technology Innovation Project of Guangdong Province (2022KJCX008).

## Data Availability Statements

All data are incorporated into the article and its online supplementary material. The data underlying this article are available in the article and in its online supplementary material.

## Supplementary Material

Web_Material_coad049
