# Supplementary material for: Energy budgets of captive Chinese pangolins (Manis pentadactyla)
Source: Conserv Physiol. 2023 Jul 13;11(1):coad049. doi: 10.1093/conphys/coad049 (PMC10348091; doi:10.1093/conphys/coad049)
Supplement: Web_Material_coad049 [file web_material_coad049.zip › Supplement file.pdf]

There are some pictures about chamber and animals.

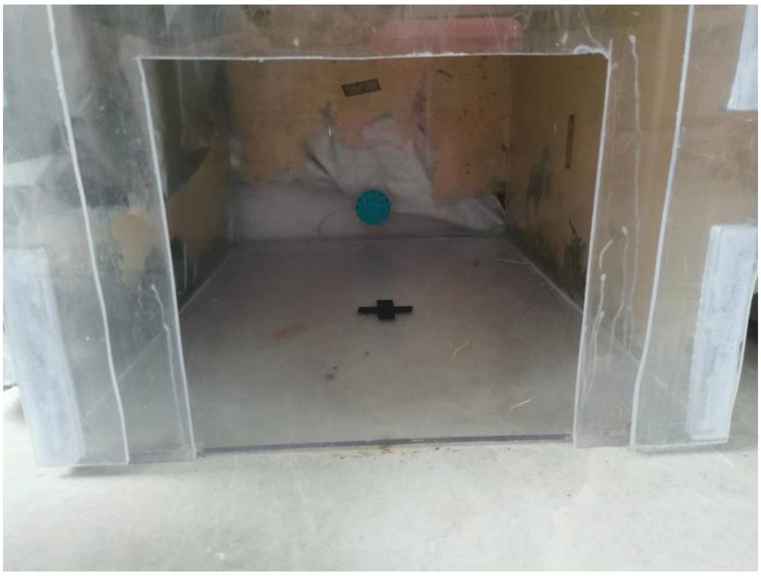

Figure S1. The chamber of resting metabolic rate.

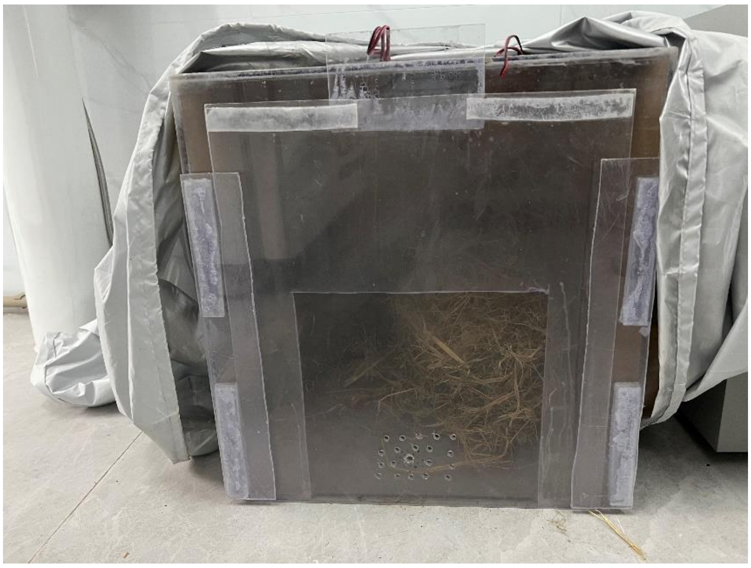

Figure S2. The chamber of resting metabolic rate.

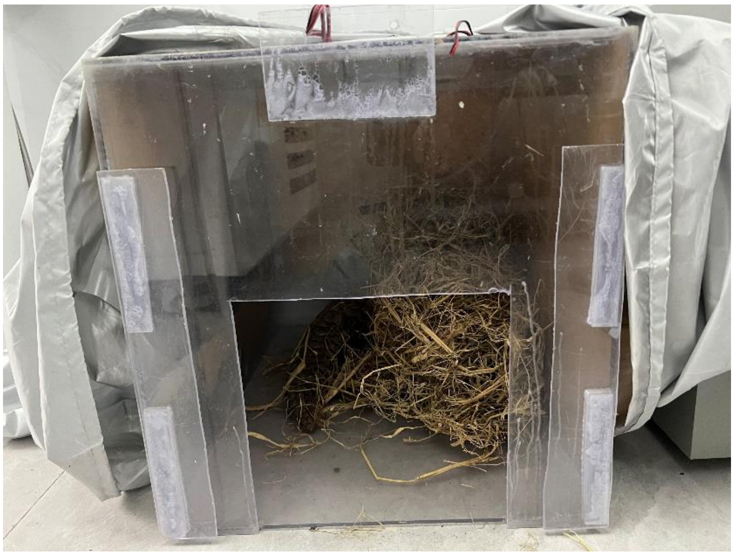

Figure S3. The chamber of resting metabolic rate.

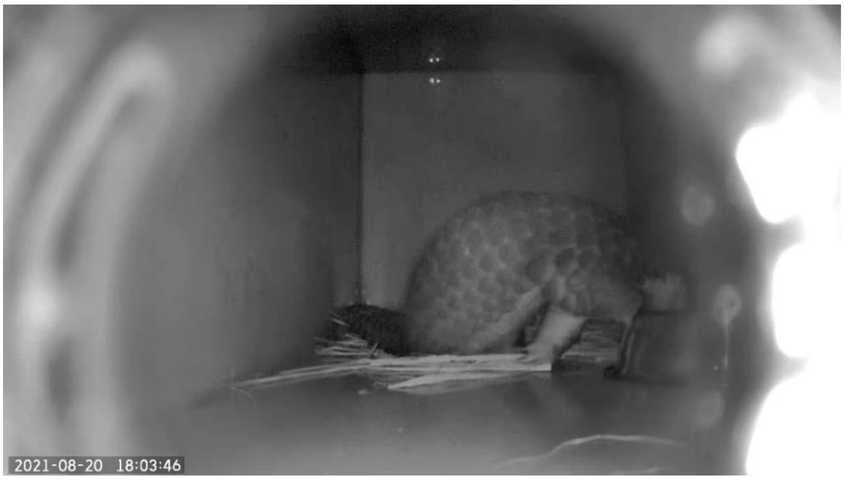

Figure S4. The chamber of daily energy expenditure.
